# Psychological determinants of COVID-19 vaccine acceptance among urban slum dwellers of Bangladesh

**DOI:** 10.3389/fpubh.2022.958445

**Published:** 2022-09-16

**Authors:** Muhammad Mainuddin Patwary, Mondira Bardhan, Sardar Al Imran, Mehedi Hasan, Faiza Imam Tuhi, Sama Jamila Rahim, Md. Navid Newaz, Mahadi Hasan, Md. Zahidul Haque, Asma Safia Disha, Md. Riad Hossain, Alfonso J. Rodriguez-Morales, Fahimeh Saeed, Sardar Khan Nazari, Sheikh Shoib

**Affiliations:** ^1^Environment and Sustainability Research Initiative, Khulna, Bangladesh; ^2^Environmental Science Discipline, Khulna University, Khulna, Bangladesh; ^3^Development Studies Discipline, Khulna University, Khulna, Bangladesh; ^4^Department of Environmental Science and Disaster Management, Bangabandhu Sheikh Mujibur Rahman Science and Technology University, Gopalganj, Bangladesh; ^5^Department of Statistics, University of Dhaka, Dhaka, Bangladesh; ^6^Institute of Disaster Management, Khulna University of Engineering and Technology, Khulna, Bangladesh; ^7^Grupo de Investigación Biomedicina, Faculty of Medicine, Fundacion Universitaria Autónoma de las Américas, Pereira, Colombia; ^8^Institución Universitaria Visión de las Américas, Pereira, Colombia; ^9^Master of Clinical Epidemiology and Biostatistics, Universidad Cientifica del Sur, Lima, Peru; ^10^School of Medicine, Universidad Privada Franz Tamayo, Cochabamba, Bolivia; ^11^Psychosis Research Center, University of Social Welfare and Rehabilitation Sciences, Tehran, Iran; ^12^Sakha Private Hospital, Kabul, Afghanistan; ^13^Department of Psychiatry, Jawahar Lal Nehru Memorial Hospital (JLNMH), Srinagar, India

**Keywords:** COVID-19, vaccine acceptance, Bangladesh, vaccine hesitancy, slum people, psychological antecedents, 5C sub-scales

## Abstract

**Introduction:**

Coronavirus disease 2019 (COVID-19) vaccination has emerged as a promising approach to counter the harmful impacts of the pandemic. Understanding the psychological components that may impact an individual's attitude toward COVID-19 vaccination is crucial for generating evidence-based ways to minimize vaccine hesitancy. This study determined the psychological antecedents regarding vaccine acceptance among urban slum people of Bangladesh.

**Methods:**

From 5 July to 5 August 5, 2021, a face-to-face survey was conducted in the urban slum of two large cities in Bangladesh. The questionnaire considered socio-demographics, health-related characteristics, psychological determinants, sources of information, and conspiracy beliefs regarding COVID-19. The 5C sub-scales were used to assess psychological antecedents. Five stepwise binary logistic regression models evaluated significant predictors for confidence, complacency, calculation, constraints, and collective responsibility. Multinomial logistic regression was used to determine the relationship between psychological antecedents and vaccine acceptability.

**Results:**

The study revealed that the slum residents with a high level of confident (89.94%), complacent (72.73%), having constraints (82.31%), calculative (84.80%), and responsible (93.30%) showed a higher vaccine acceptance rate. Higher vaccine acceptance was related to the believer in natural-made origin (85.96%) and those who rejected anti-vaccination (88.44%). The information acquired from newspapers differed significantly (*p* < 0.05), though TV or radio was the most common primary information source about COVID-19 vaccines (74.75%). The regression result revealed that marital status, education, family income, and perceived health condition were significantly associated with the 5C domains. Two psychological antecedents including complacency (OR = 3.97; *p* < 0.001) and collective responsibility (OR = 0.23; *p* < 0.001) were significantly associated with vaccine acceptance.

**Conclusions:**

Different predictors significantly affect psychological antecedents related to COVID-19 vaccine uptake. Therefore, considering the factors, targeted actions based on the findings may help to lower vaccine reluctance and boost vaccination rates.

## Introduction

Vaccines are a material used to stimulate the development of antibodies and confer immunity against existing and emerging infectious diseases (1). Vaccines are a miracle of modern medicine. More lives have been saved due to them than any other human invention (2). The novel coronavirus disease known as COVID-19 was first detected in Wuhan, China, in late December 2019. With the rapid transmission rate, this virus spread worldwide soon thereafter. Consequently, the World Health Organization (WHO) proclaimed COVID-19 a global pandemic on 11 March 2020 (3). As of 16 March 2022, the world has experienced a catastrophic situation due to the coronavirus disease (COVID-19) that resulted in more than 460 million cases and around 6 million deaths across 220 countries (4). Since SARS-CoV-2 is a highly infectious virus that affects people worldwide, vaccines are the most significant public health intervention and the most effective technique for protecting the population against coronavirus disease 2019 (COVID-19) (5, 6).

Considering the catastrophic scenario, vaccinations are one of the most crucial public health interventions for limiting the spread of dangerous infections and their damage. The WHO estimates that vaccines have saved at least 10 million lives throughout the globe (7, 8). Vaccination helps to develop antibodies and provide immunity against the virus, which has been shown to reduce pandemic severity by reducing COVID-19 infection, hospitalization, and mortality. According to a recent study, when people's immunity reaches 67%, there is a possibility to decline in COVID-19 infections (9). It is impressive that numerous viable COVID-19 vaccines have developed in less than a year (10). Scientific and pharmaceutical companies have developed dozens of COVID-19 vaccines, including Pfizer–BioNTech, Moderna, Janssen, Sinopharm-BBIBP, Sputnik V, CoviVac, and Covaxin, to protect humans (11). However, the protection of the world's population depends on the availability of vaccine dosages and government immunization programs (10). A report demonstrated that by mid-March 2021, 380 million doses of COVID-19 vaccination had been distributed worldwide. However, the report showed that the vaccine acceptance tendency worldwide is still lagging (12). By the end of 2021, the European Union intends to have vaccinated 70% of its adult population. More than 51 million vaccine doses had been provided across the EU as of the end of 2021, with Denmark and Spain having the highest vaccination rates (13). Several high-income countries (HICs) have made significant progress, with Israel leading the way, having vaccinated half of its population by the end of February (14). However, many HICs have found it challenging to get COVID-19 vaccines due to vaccine hesitancy (11, 15). As HICs began vaccinating, new administrative issues arose, and new methods were offered to address supply hurdles, such as extending the interval between vaccine doses. On the other hand, despite their extensive expertise from the Expanded Programme on Immunization (EPI), which began in 1974, lower-middle-income countries (LMICs) may confront more extra problems than HICs (16).

A successful vaccination program depends on the extent of people's willingness to accept the vaccine, the demand for the vaccine, and their behavior toward vaccination (17, 18). However, increasing hesitancy toward vaccination limits the success of a vaccination program (19, 20); such hesitancy is defined by the delay in accepting the available vaccine (21). The WHO labeled vaccine hesitancy as a serious public health threat that raised concern about the successful implementation of vaccination worldwide (22). As seen in the 2018 measles epidemic in New York City, vaccination reluctance led to continuous transmission (23). Rapid development of vaccines, conspiracy theories on vaccine origin, lack of trust in government, and religious misconceptions have been identified as major obstacles to vaccine hesitancy (24). Vaccine reluctance is context-dependent and impacted by time, location, and vaccines, as well as psychological variables (25). Studies suggest that individual attitudes regarding vaccination, in general, and COVID-19 immunization, in particular, appear to be influenced by psychological variables. This is mostly attributable to the psychological impacts of the current pandemic, which was accompanied by a deluge of information (16, 26). Therefore, it is important to analyze the psychological aspects of vaccination to determine the individual behavior toward vaccination, which might help in the development of evidence-based strategies to minimize vaccine reluctance.

Grounded on theories of vaccine hesitancy and acceptance, Betsch et al. developed and validated a vaccination tool (5C model) to explain psychological behavior toward vaccination (27). The 5C scale offers a reliable and psychologically sound approach for tracking vaccination behavior around the globe. The researchers used the 5C scale to study how anticipatory elements affect vaccination behavior as well as the deep understanding of how each person's mental depictions, attitude, and behavioral propensities are influenced by their surrounding environment and contexts. The 5C scale consisted of five psychological antecedents, including confidence, complacency, constraints, calculation, and collective responsibility (27, 28). Currently, these antecedents are widely used in high-income countries to assess vaccine hesitancy to determine the vaccination uptake rates (29). Several studies reported the psychological antecedents of the COVID-19 vaccine among different population groups in different countries, including Bangladesh (30–33); however, there is no study assessing the psychological determinants of vaccination among socioeconomically disadvantaged people using 5C scale.

Early on, there were conspiracy beliefs about the origins of the COVID-19 pandemic. These opinions were based on the idea that the virus was created by humans (34). These bad ideas also included thoughts about future vaccinations, such as charges of vaccination-enforcement conspiracies, which would be used to implant microchips in individuals to control people. Further, social media users have expressed concern about suggestions that COVID-19 vaccines could cause infertility and limit the human population increase (34, 35). Such unverified information is frequently disseminated on uncontrolled social media and other news media platforms, which might significantly influence the individual decision toward vaccination (30). Earlier studies also showed a significant correlation between conspiracy beliefs and vaccine hesitancy (30, 36).

In Bangladesh, more than 2 million people live in urban slum areas (37). Slums are characterized by inadequate healthcare services, limited educational options, limited living space, and a dearth of employment prospects (38). Being historically poor healthcare systems in Bangladesh (39), the COVID-19 pandemic compounded the plight of urban slum inhabitants who were already suffering financially and lacked access to healthcare services due to inequitable services and economics (40).

Data suggests that 75% of the slum population lives in a single room, and 45% of them suffer from infectious and parasitic diseases regularly (40), whereas only 13.9% are able to seek healthcare services from formal healthcare professionals (41). On top of that, COVID-19 has brought an additional burden to them. A study reported that slum populations are more vulnerable to COVID-19 infection than others and experience higher morbidity (42). In this situation, the slum-dwellers possible reluctance to take the COVID-19 vaccination might render them more susceptible to the virus.

Vaccine uptake determines the extent to which the population is sufficiently protected, which may vary across sub-populations such as the slum population, the ethnic minority population, and healthcare workers (43, 44). There have been a couple of studies conducted so far to determine the COVID-19 vaccine acceptance among the general population (18, 32, 45) and healthcare professionals (43) in Bangladesh. However, all these studies investigated the vaccination rates of well-educated and privileged citizens in Bangladesh. Another study in Bangladesh focused on the vaccination status of the low-income population (46); however, this study did not consider any empirical model to predict vaccination behavior. Further, none of the studies evaluated the impact of conspiracy beliefs on individual vaccination behavior. Thus, this study determined the prevalence of psychological antecedents and their associated factor toward COVID-19 vaccination using the 5C scale among urban slum dwellers in Bangladesh. The major objectives of this study were (a) to assess the psychological antecedents of COVID-19 vaccination and the factors associated with 5C domains and (b) the effect of embracing COVID-19 vaccine conspiracy beliefs on vaccine acceptance among the urban slum population in the country. Other minor objective was assessing the role of information sources in COVID-19 vaccination.

## Methods

### Study settings and participants

**Figure 1:**
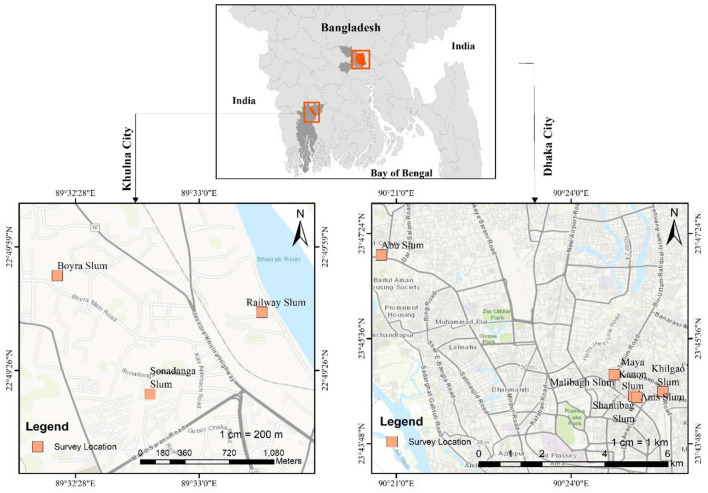
Study survey location.

A cross-sectional survey design was employed in this study. A face-to-face survey was conducted in Bangladesh between 5 July and 5 August 2021, amid a devastating second wave of infections before the widespread vaccine was available. Individuals aged at least 18 years old without receiving their first dose of COVID-19 vaccine in urban slums in Bangladesh were included. Using a simple random sampling technique, the data were collected from urban slums (location shown in Figure 1) in Dhaka and Khulna city of Bangladesh.

Since no previous studies were available that suit our study measures, an online calculator was adopted to estimate the sample size for our research. As determined by the sample size calculator (https://statulator.com/ accessed on July 1, 2021), the minimum number of respondents is 385. The calculation were based on a 10% non-response rate, 5% precision, a 50% proportion, and a 95% confidence range for the overall slum population estimate of 2.2 million (37). Therefore, we collected 410 sample respondents from slums of two cities in Bangladesh. However, 10 participants were eliminated from the study due to prior vaccination against COVID-19. After excluding them, the final study contained 400 respondents, including 169 males and 231 females. Before completing the survey, all participants electronically consented. Therefore, participants were not needed to complete the form in its entirety. This survey did not require participants to provide their names or email addresses, ensuring that respondents could not be identified individually. Further, the research ethical clearance board of the Institute of Disaster Management, Khulna University of Engineering & Technology, Khulna, Bangladesh, approved this study.

### Measures

A structured questionnaire was developed and sent to each respondent to gather data. The questionnaire elicited information on their sociodemographic and health-related features, intentions to receive a COVID-19 vaccine, 5C psychological antecedents, information sources, and conspiracy beliefs surrounding COVID-19.

#### Psychological antecedents

The decision to vaccinate is influenced by several factors, some of which are out of the control of the individual (such as a parent) and others within their control. There are five antecedents such as confidence, complacency, constraints, calculation, and collective responsibility, comprised of a 5C scale that determines the psychological factors of vaccination. Five psychological antecedents of vaccination are evaluated using the 5C scale, which sheds light on how the respondent's particular environment and context shape their distinctive mental representations, attitudes, and behaviors (27). The 5C scale consists of a ten-item scale (involving two items for each determinant). These items were chosen following a prior methodology established by Betsch et al. (27). The following items were used to measure confidence: (1) I am confident that public authorities decide in the best interest of the community; (2) I am entirely confident that the COVID-19 vaccine is effective. The following items were used to measure complacency: (1) It is unnecessary to get vaccinated as it cannot prevent COVID-19; (2) My immune system is robust, which protects me. The following elements were used to evaluate constraints: (1) Everyday work stress may prevent me from getting vaccinated; (2) Visiting the doctor makes me feel uncomfortable; this keeps me from being vaccinated. The calculation was evaluated based on the following criteria: (1) When I get vaccinated, I will consider whether it is effective or not; (2) Before I get vaccinated, I need to know about the details of the vaccine. Finally, the following items were used to measure collective responsibility: (1) I will take the vaccine, in that the weaker immune people will get protection; (2) COVID-19 vaccination is a collective action to prevent the spread of disease.

#### Source of information and conspiracy belief

Respondents were asked about the essential information sources they adopted for vaccine information. The following sources were designed as options: Social media, TV/Radio, Newspapers, Doctors/nurse/community healthcare staff, Friends/Family members, and Neighbors.

Respondent's conspiracy belief on COVID-19 and vaccine was assessed using two questions following (30). The first question was, “Do you oppose vaccination altogether?.” Responses were collected as Yes, No, or No opinions. The second question was, “What is the belief about the origin of human coronavirus?.” Again, responses were recorded as whether COVID-19 was naturally made from animals, manufactured, and part of a conspiracy plot and no opinion.

#### Willingness to accept the vaccine

A single question was used to assess the participant's willingness to receive a COVID-19 vaccination. Respondents were asked, “Will you take the Covid-19 vaccination when it becomes available?.” The possible answer options were “Yes,” “No,” or “Not sure.” Participants were divided into three groups: those who planned to take the vaccine (response = “Yes”), those who were unsure (response = “Not sure”), and those who were opposed to receiving the vaccine (response = “No”).

#### Sociodemographic and health variables

Sociodemographic variables included gender, age, marital status, education, occupation, family type, and monthly income. Gender was assessed by asking whether male or female. Age was a continuous measure. Respondents were asked about their education level using four bins: (1) no formal education, (2) currently primary level, (2) Secondary School Certificate (SSC) level, or (3) college or higher degree. Respondents classified the family type as currently they live in a nuclear or joint family. The respondent ranked their occupation as unemployed, student or worker, day laborer, small business, or housewife. Finally, monthly income was assessed by asking for their monthly family income on ≤ 5,000 BDT (<58 US$), 5,001–10,000 BDT (58–115 US$), 10,001–15,000 BDT (116–173 US$), and > 15,000 BDT (>173 US$).

The health-related variables were COVID-19 test positivity, body mass index (BMI), having any long-standing illness (es), perceived health status, smoking habit, and childhood vaccination status. The COVID-19 susceptibility, presence of the long-standing condition, smoking habit, and childhood vaccination status were assessed by asking a respondent to indicate Yes or No. Body Mass Index (BMI) was calculated with the respondent's height (m^2^) and weight (kg). The respondent's perceived health status was evaluated by asking them 5-items, including very good, good, fair, bad, and very bad.

### Data analysis

Participants were separated into three groups according to their vaccination intentions: those who agreed to get the vaccine, those who were unsure, and those who were opposed to receiving the vaccine. The latter two categories have been merged as “undecided/unwilling.” We selected two groups rather than three when doing statistical analysis on vaccination intentions to underline the possibility of differentiation between individuals who planned to accept a COVID-19 vaccine and those who did not to uncover characteristics that indicated one's desire to vaccinate. For categorical variables, Chi-square tests were employed, and Kruskal-Wallis tests were used for continuous variables. Additionally, a Chi-square test was used to examine the relationship between the sources of information, conspiracy beliefs, and vaccination intention.

Pairwise correlations between category variables were estimated using a chi-square test. The respondent's “Yes” or “No” status was determined based on their average 5C score at the cut-off points. We used five stepwise binary logistic regression models including all variables to identify the most significant factors influencing levels of confidence, complacency, calculation, constraints and collective responsibility. Statistical significance was defined as a *p* value of less than 0.05, and results were provided as odds ratios (OR) with 95% confidence intervals (CI). Additionally, multinomial logistic regression was used to examine the relationship between the 5C domains and willingness to receive the COVID-19 vaccination, adjusting for sociodemographic and health characteristics. To assess the effectiveness of 5C subscales in predicting COVID-19 vaccination hesitancy, we calculated the area under the receiver operator characteristic (ROC) curve (AUC).

## Results

### Sociodemographic characteristics

Table 1 summarizes the baseline characteristics of our study population. Out of 400 samples, 227 (56.8%) were female respondents. The mean age of the total sample was 33.43 (±11.25) years. Of the total, about 90% (*n* = 360) were married. The majority had no formal education (52.5%, *n* = 210). Most participants were day labor (29%, *n* = 116). Around 91.8% (*n* = 367) belonged to a nuclear family. About half of the participants (43.5%, *n* = 174) had a monthly family income between 5,000–10,000 BDT (US$ 58-115). More than 90% (*n* = 369) of the participants were not diagnosed with COVID-19. The mean BMI was 22.50 (±3.61). The majority (64.2%, *n* = 257) of respondents did not have a long-standing illness, and 34.5% (n = 138) reported that their health status was good. The majority (68.8%, *n* = 275) of respondents reported as being non-smokers. Around 81.1% (*n* = 327) participated in their childhood vaccination.

**Table 1:** Descriptive statistics of respondents' intention to get vaccinated against COVID-19 (*N* = 400).

**Variables**	**Frequency (*N*)**	**%**
**Sociodemographic characteristics**		
**Gender**		
Male	173	43.2
Female	227	56.8
**Age**	33.43 (±11.25)	
**Marital status**		
Single	35	8.8
Married	360	90.0
Divorced	5	1.2
**Education**		
No formal education	210	52.5
Primary level	115	28.7
SSC	50	12.5
≥College	25	6.3
**Occupation**		
Unemployed	44	11.0
Student	14	3.5
Worker	106	26.5
Day labor	116	29.0
Small business	31	7.8
Housewife	89	22.2
**Family type**		
Nuclear	367	91.8
Joint	33	8.2
**Monthly family income (BDT)**		
≤ 5,000 (US$ <58)	90	22.5
5,001–10,000 (US$ 58–115)	174	43.5
10,001–15,000 (US$ 115–173)	89	22.2
>15,000 (US$ <173)	47	11.8
**Health-related characteristics**		
**Tested positive for COVID-19**		
No	369	92.2
Yes	31	7.8
**BMI**	22.50 (±3.61)	
**Long-standing illness(es)**		
No	257	64.2
Yes	143	35.8
**Perceived health condition**		
Very good	133	33.3
Good	138	34.5
Fair	86	21.5
Bad	26	6.5
Very bad	17	4.2
**Smoking**		
No smoking	275	68.8
Current smoker	108	27.0
Former smoker	17	4.2
**Childhood vaccination(s)**		
No	73	18.2
Yes	327	81.8

### Prevalence of psychological antecedents of vaccination

**Figure 2:**
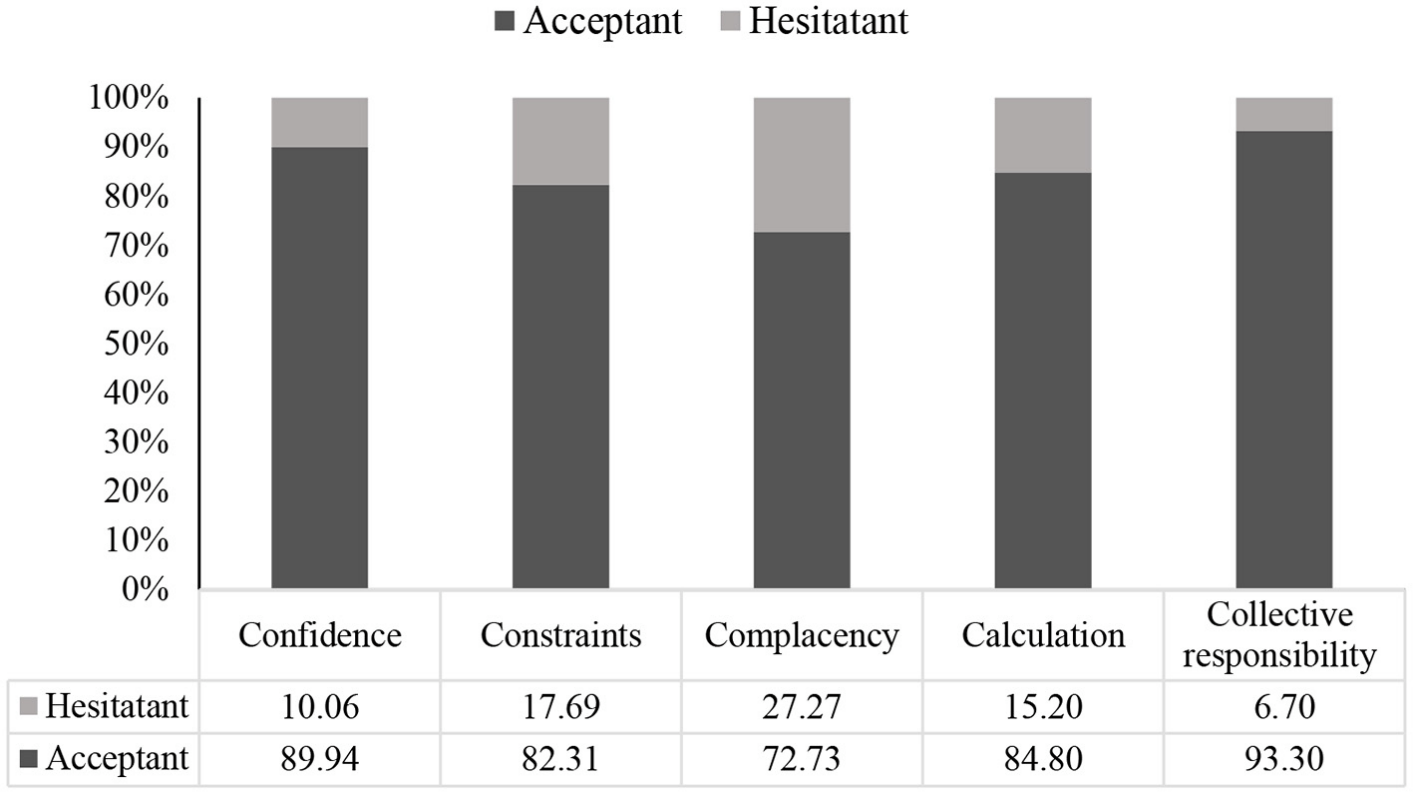
Psychological antecedents for acceptance of the vaccine.

Figure 2 illustrates the psychological antecedents of vaccine acceptance among slum dwellers. Approximately 90% of respondents who said “yes” to vaccine acceptance showed confidence (*p* < 0.001, χ^2^ test = 13.16) regarding COVID-19 vaccination and its effectiveness. About 72.73% were complacent (*p* < 0.001, χ^2^ test = 26.67), 84.80% calculated the effectiveness and detailed information of vaccine (*p* > 0.05, χ^2^ test = 3.30), and 93.30% respondents showed collective responsibility for accepting vaccines (*p* < 0.001, χ^2^ test = 38.54). However, 82.31% faced constraints regarding vaccination, though they were optimistic about getting vaccinated (*p* > 0.05, χ^2^ test = 0.15).

### The information source of the COVID-19 vaccine and its relation to willingness to accept the vaccine

**Figure 3:**
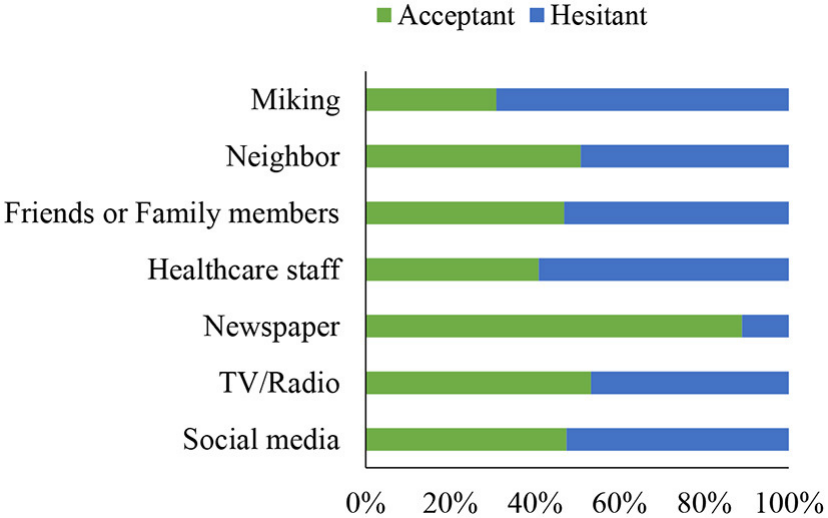
Information sources among the acceptant and hesitant groups.

Figure 3 illustrates the information source distribution among the vaccine acceptant and hesitant groups. TV or radio was reported as the most common primary source of information about COVID-19 vaccines (*n* = 299, 74.75%), followed by neighbors (*n* = 259, 64.75%), friends or family members (*n* = 228, 57%), social media (*n* = 116, 29 %), healthcare staff (*n* = 41, 10.25%), newspaper (*n* = 37, 9.25%), and miking (*n* = 6, 1.5%). Individuals who declined COVID-19 vaccination were more likely to rely on friends or family (55.66 vs. 63.01%) for vaccine information; however, the differences were not statistically significant (*p* = 0.351, χ^2^ test). In contrast, differences in information obtained through newspapers were significant (*p* = 0.012, χ^2^ test).

### Conspiracy belief on COVID-19 origin and altogether anti-vaccination and its relation to vaccine acceptance

**Figure 4:**
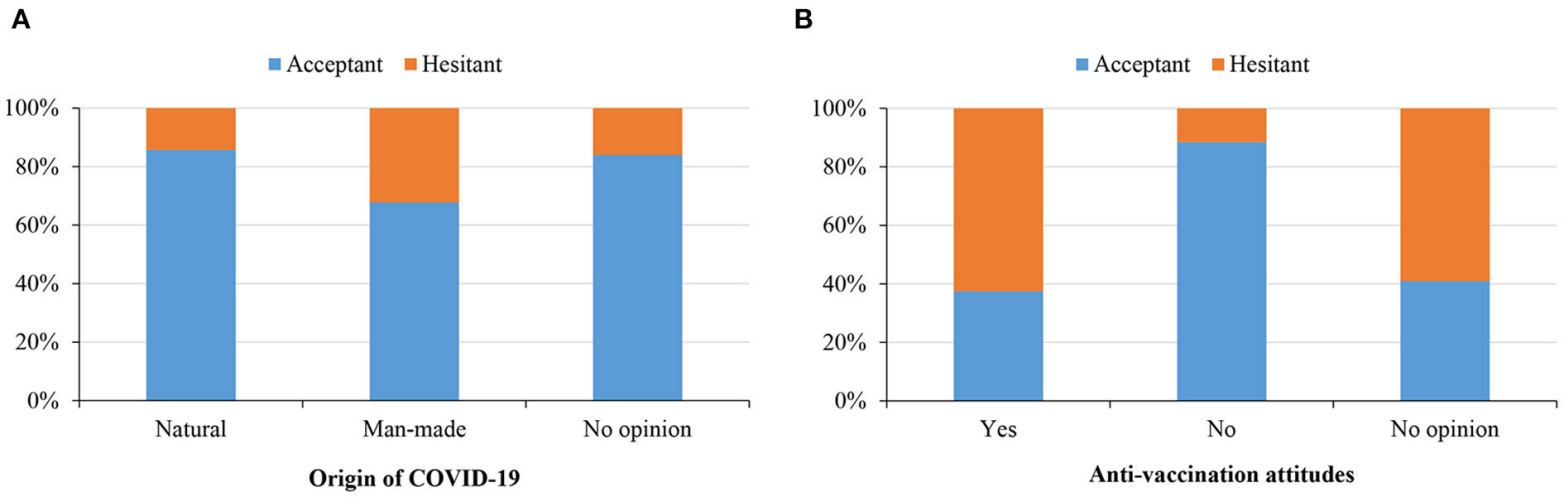
Willingness to accept vaccine based on the belief in COVID-19 origin **(A)** and attitude toward anti-vaccination **(B)**.

Figure 4A demonstrates the vaccine acceptance rate based on the conspiracy belief toward COVID-19 origin. Of the total sample, 15.5% (*n* = 62) believed that SARS-CoV-2 had a human-made origin, while 17.5% (*n* = 70) believed in the natural source of the virus. However, a major portion reported no opinion (*n* = 268, 67%). Additionally, believing in a naturally occurring source of the virus was significantly associated (*p* = 0.008; χ^2^ test) with a high intention to receive the COVID-19 vaccine compared to those who believed in a manufactured source of the virus and those who had no opinion on the virus's origin (85.71% vs. 67.74% vs. 83.96%).

Figure 4B illustrates the vaccine rates among the respondents based on respondents' attitudes toward altogether anti-vaccination. Only 37.50% of anti-vaccination participants (*n* = 12) reported an intention to receive COVID-19 vaccination, compared to 40.91% in the “no opinion” group (*n* = 9) and 88.44 % among those who rejected anti-vaccination (*n* = 306, *p* < 0.001; χ^2^ test).

### Univariate analysis of 5C domains with independent variables

Table 2 demonstrates the univariate analysis of the 5C domain individually predicted by the independent variables. Education level (*p* < 0.01), monthly family income (*p* < 0.05), and perceived health condition (*p* < 0.05) significantly affected the confidence regarding vaccination. Further, the COVID-19 related constraints were significantly affected by gender (*p* < 0.01), education level (*p* < 0.01), occupation status (*p* < 0.01), monthly family income (*p* < 0.001), and smoking habit (*p* < 0.01) of the participants.

**Table 2:** Univariate analysis of 5C domains.

**Variables**	**Confidence**			**Constraints**			**Complacency**			**Calculation**			**Collective responsibilitys**		
	***N*** **(%)**			***N*** **(%)**			***N*** **(%)**			***N*** **(%)**			***N*** **(%)**		
	**Yes**	**No**	***p*-value**	**Yes**	**No**	***p*-** **value**	**Yes**	**No**	***p*- value**	**Yes**	**No**	***p*-** **value**	**Yes**	**No**	***p*- value**
**Sociodemographic characteristics**															
**Gender**			0.147			0.002^**^			0.976			0.967			0.104
Male	66 (39.1)	103 (60.9)		127 (48.8)	46 (32.9)		95 (43.2)	78 (43.3)		88 (43.3)	85 (43.1)		98 (47.1)	75 (39.1)	
Female	107 (46.3)	124 (53.7)		133 (51.2)	94 (67.1)		125 (56.8)	102 (56.7)		115 (56.7)	112 (56.9)		110 (52.9)	117 (60.9)	
**Age**	31.75 ± 11.06	34.65 ± 11.26	0.192	34.01 ± 10.76	32.34 ± 12.07	0.052	34.75 ± 11.10	31.81 ± 11.25	0.119	33.38 ± 11.46	33.47 ± 11.06	0.239	32.38 ± 10.71	34.56 ± 11.73	0.769
**Marital status**			0.955			0.105			0.503			0.047^*^			0.897
Single	14 (8.3)	21 (9.1)		19 (7.3)	16 (11.4)		20 (9.1)	15 (8.3)		24 (11.8)	11 (5.6)		19 (9.1)	16 (8.3)	
Married	153 (90.5)	207 (89.6)		236 (90.8)	124 (88.6)		196 (89.1)	164 (91.1)		176 (86.7)	184 (93.4)		186 (89.4)	174 (90.6)	
Divorced	2 (1.2)	3 (1.3)		5 (1.9)	0 (0.0)		4 (1.8)	1 (0.6)		3 (1.5)	2 (1.0)		3 (1.4)	2 (1.0)	
**Education**			0.004^**^			0.001^**^			0.000^***^			0.382			0.041^*^
No formal education	71 (42.0)	139 (60.2)		155 (59.6)	55 (39.3)		137 (62.3)	73 (40.6)		99 (48.8)	111 (56.3)		96 (46.2)	114 (59.4)	
Primary level	59 (34.9)	56 (24.2)		65 (25.0)	50 (35.7)		52 (23.6)	63 (35.0)		64 (31.5)	51 (25.9)		64 (30.8)	51 (26.6)	
SSC	27 (16.0)	23 (10.0)		28 (10.8)	22 (15.7)		24 (10.9)	26 (14.4)		25 (12.3)	25 (12.7)		32 (15.4)	18 (9.4)	
≥ College	12 (7.1)	13 (5.6)		12 (4.6)	12 (9.3)		7 (3.2)	18 (10.0)		15 (7.4)	10 (5.1)		16 (7.7)	9 (4.7)	
**Occupation**			0.124			0.001^**^			0.015^*^			0.129			0.025^*^
Unemployed	18 (10.7)	44 (11.0)		28 (10.8)	16 (11.4)		32 (14.5)	12 (6.7)		24 (11.8)	20 (10.2)		19 (9.1)	25 (13.0)	
Student	5 (3.0)	14 (3.5)		6 (2.3)	8 (5.7)		5 (2.3)	9 (5.0)		6 (3.0)	8 (4.1)		8 (3.8)	6 (3.1)	
Worker	47 (27.8)	106 (26.5)		61 (23.5)	45 (32.1)		61 (27.7)	45 (25.0)		64 (31.5)	42 (21.3)		66 (31.7)	40 (20.8)	
Day labor	38 (22.5)	116 (29.0)		94 (36.2)	22 (15.7)		68 (30.9)	48 (26.7)		52 (25.6)	64 (32.5)		56 (26.9)	60 (31.3)	
Small business	17 (10.1)	31 (7.8)		21 (8.1)	10 (7.1)		12 (5.5)	19 (10.6)		18 (8.9)	13 (6.6)		21 (10.1)	10 (5.2)	
Housewife	44 (26.0)	89 (22.3)		50 (19.2)	39 (27.9)		42 (19.1)	47 (26.1)		39 (19.2)	50 (25.4)		38 (18.3)	51 (26.6)	
**Family type**			0.279			0.331			0.298			0.649			0.760
Nuclear	158 (93.5)	209 (90.5)		236 (90.8)	131 (93.6)		199 (90.5)	168 (93.3)		185 (91.1)	182 (92.4)		190 (91.3)	177 (92.2)	
Joint	11 (6.5)	22 (9.5)		24 (9.2)	9 (6.4)		21 (9.5)	12 (6.7)		18 (8.9)	15 (7.6)		18 (8.7)	15 (7.8)	
**Monthly family income**			0.033^*^			<0.001^***^			<0.001^***^			0.314			<0.001^***^
≤ 5,000	33 (19.5)	57 (24.7)		70 (26.9)	20 (14.3)		64 (29.1)	26 (14.4)		38 (18.7)	52 (26.4)		37 (17.8)	53 (27.6)	
5,001–10,000	71 (42.0)	103 (44.6)		123 (47.3)	51 (36.4)		100 (45.5)	74 (41.1)		93 (45.8)	81 (41.1)		77 (37.0)	97 (50.5)	
Alone 10,001–15,000	36 (21.3)	53 (22.9)		42 (16.2)	47 (33.6)		35 (15.9)	54 (30.0)		46 (22.7)	43 (21.8)		59 (28.4)	30 (15.6)	
>15,000	29 (17.2)	18 (7.8)		25 (9.6)	22 (15.7)		21 (9.5)	26 (14.4)		26 (12.8)	21 (10.7)		35 (16.8)	12 (6.3)	
**Health-related characteristics**															
**Tested positive for COVID-19**			0.678			0.217			0.252			0.784			0.482
No	157 (92.9)	212 (91.8)		243 (93.5)	126 (90.0)		206 (93.6)	163 (90.6)		188 (92.6)	181 (91.9)		190 (91.3)	179 (93.2)	
Yes	12 (7.1)	19 (8.2)		17 (6.5)	14 (10.0)		14 (6.4)	17 (9.4)		15 (7.4)	16 (8.1)		18 (8.78)	13 (6.8)	
**BMI**	22.70 ± 4.21	22.35 ± 3.11	0.582	22.77 ± 3.74	22.00 ± 3.33	0.123	22.74 ± 3.64	22.22 ± 3.56	0.450	22.33 ± 4.05	22.68 ± 3.10	0.283	22.43 ± 4.09	22.57 ± 3.02	0.410
**Long-standing illness(es)**			0.253			0.818			0.362			0.743			0.003^**^
No	114 (67.5)	143 (61.9)		166 (63.8)	91 (65.0)		137 (62.3)	120 (66.7)		132 (65.0)	125 (63.5)		148 (71.2)	83 (43.2)	
Yes	55 (32.5)	88 (38.1)		94 (36.2)	49 (35.0)		83 (37.7)	60 (33.3)		71 (35.0)	72 (36.5)		60 (28.8)	109 (56.8)	
**Perceived health condition**			0.031^*^			0.881			0.024^*^			0.889			0.001^**^
Very good	66 (39.1)	67 (29.0)		90 (34.6)	43 (30.7)		73 (32.3)	60 (33.3)		67 (33.0)	66 (33.5)		83 (39.9)	50 (26.0)	
Good	63 (37.3)	75 (32.5)		87 (33.5)	51 (36.4)		63 (28.6)	75 (41.7)		71 (35.0)	67 (34.0)		76 (36.5)	62 (32.3)	
Fair	27 (16.0)	59 (25.5)		55 (21.2)	31 (22.1)		54 (24.5)	32 (17.8)		46 (22.7)	40 (20.3)		37 (17.8)	49 (25.5)	
Bad	7 (4.1)	19 (8.2)		18 (6.9)	8 (5.7)		17 (7.7)	9 (5.0)		12 (5.9)	14 (7.1)		8 (3.8)	18 (9.4)	
Very bad	6 (3.6)	11 (4.8)		10 (3.8)	7 (5.0)		13 (5.9)	4 (2.2)		7 (3.4)	10 (5.1)		4 (1.9)	13 (6.8)	
**Smoking**			0.116			0.001^**^			0.873			0.073			0.110
No smoking	123 (72.8)	152 (65.8)		162 (62.3)	113 (80.7)		149 (67.7)	126 (70.0)		144 (70.9)	131 (66.5)		136 (65.4)	139 (72.4)	
Current smoker	37 (21.9)	71 (30.7)		84 (32.3)	24 (17.1)		61 (27.7)	47 (26.1)		47 (23.2)	61 (31.0)		65 (31.3)	43 (22.4)	
Former smoker	9 (5.3)	8 (3.5)		14 (5.4)	3 (2.1)		10 (4.5)	7 (3.9)		12 (5.9)	5 (2.5)		7 (3.4)	10 (5.2)	
**Childhood vaccination (s)**			0.825			0.674			0.630			0.117			0.071
No	30 (17.8)	43 (18.6)		49 (18.8)	24 (17.1)		42 (19.1)	31 (17.2)		31 (15.3)	42 (21.3)		31 (14.9)	42 (21.9)	
Yes	139 (82.2)	188 (81.4)		211 (81.2)	116 (82.9)		178 (80.9)	149 (82.8)		172 (84.7)	155 (78.7)		177 (85.1)	150 (78.1)	

* *p* < 0.05,

** *p* < 0.01,

*** *p* < 0.001. Significant coefficients are shown in bold.

The complacency domain was significantly anticipated by education level (*p* < 0.001), occupation status (*p* < 0.05), monthly family income (*p* < 0.001), and perceived health condition (*p* < 0.05). The collective responsibility was significantly predicted by the education level *(p* < 0.05), occupation status (*p* < 0.05), monthly family income (*p* < 0.001), long-standing illness (*p* < 0.05), and perceived health condition (*p* < 0.01), where only marital status was significantly related to the calculation domain (*p* < 0.05).

### Predictors affecting the psychological vaccination antecedents

Table 3 presents the significant predictors affecting the psychological vaccination antecedents. Monthly family income (>15,000 BDT) was a significant predictor related to the confidence antecedent (OR = 0.42; 95% CI: 0.20–0.89). Having a monthly family income between 5,001–10,000 BDT (US$ 58–115) (OR = 0.25; 95% CI: 0.12–0.50) and having a monthly family income >15,000 BDT (>US$ 173) (OR = 0.26; 95% CI: 0.11–0.62) were significantly associated with vaccination constraints. The significant complacency antecedent predictors were: primary level of education (OR = 0.55; 95% CI: 0.33–0.1), college or higher level of education (OR = 0.29; 95% CI: 0.09–0.96), and having 10,001–15,000 BDT (US$ 115–173) family income (OR = 0.35; 95% CI: 0.17–0.69). Being married was a significant predictor for the calculation domain (OR = 0.43; 95% CI: 0.20–0.91). The significant collective responsibility predictors were: monthly family income (>15,000 BDT, > US$173) (OR = 3.18; 95% CI: 1.34–7.54) and who perceived health condition was fair (OR = 0.47; 95% CI: 0.24–0.94).

**Table 3:** Factors affecting the psychological antecedents (*N* = 400).

**Predictors**	**Confidence**	**Constraints**	**Complacency**	**Calculation**	**Collective responsibility**
	**OR (95% CI)**	**OR (95% CI)**	**OR (95% CI)**	**OR (95% CI)**	**OR (95% CI)**
**Sociodemographic characteristics**					
**Gender**					
Male		Ref.			
Female		0.73 (0.36–1.47)			
**Age**					
**Marital status**					
Single				Ref.	
Married				0.43^*^(0.20–0.91)	
Divorced				0.81 (0.79–0.14)	
**Education**					
No formal education	Ref.	Ref.	Ref.		Ref.
Primary level	0.72 (0.32–1.73)	0.61 (0.36–1.04)	0.55^*^(0.33–0.1)		1.08 (0.65–1.80)
SSC	1.39 (0.57–3.39)	0.71 (0.39–1.48)	0.76 (0.38–1.57)		1.49 (0.70–3.16)
≥ College	1.40 (0.52–3.74)	0.60 (0.19–1.86)	0.29^*^(0.09–0.96)		1.65 (0.49–5.45)
**Occupation**					
Unemployed		Ref.	Ref.		Ref.
Student		0.76 (0.16–3.63)	0.57 (0.12–2.84)		0.76 (0.15–3.78)
Worker		1.02 (0.46–2.26)	0.75 (0.32–1.73)		1.92 (0.86–4.26)
Day labor		2.08 (0.89–4.82)	0.58 (0.26–1.31)		1.13 (0.52–2.46)
Small business		2.25 (0.75–6.75)	0.46 (0.15–1.31)		1.10 (0.37–3.30)
Housewife		01.21 (0.52–2.83)	0.45 (0.19–1.03)		0.78 (0.34–1.76)
**Monthly family income**					
≤ 5,000	Ref.	Ref.	Ref.		**Ref**.
5,001–10,000	0.51 (0.23–1.11)	0.61 (0.32–1.14)	0.59 (0.33–1.06)		0.96 (0.52–1.59)
10,001–15,000	0.55 (0.27–1.10)	0.25^***^(0.12–0.50)	0.35^**^(0.17–0.69)		1.94 (0.99–3.81)
>15,000	0.42^*^(0.20–0.89)	0.26^**^(0.11–0.62)	0.48 (0.21–1.09)		3.18^**^(1.34–7.54)
**Health-related characteristics**					
**Long-standing illness(es)**					
No					Ref.
Yes					0.97 (0.55–1.71)
**Perceived health condition**					
Very good	Ref.		Ref.		Ref.
Good	1.33 (0.44–4.02)		0.67 (0.68–1.12)		0.66 (0.39–1.13)
Fair	1.12 (0.37–3.38)		1.10 (0.60–2.00)		0.47^*^(0.24–0.94)
Bad	0.69 (0.22–2.15)		0.96 (0.38–2.48)		0.35 (0.12–1.01)
Very bad	0.53 (0.14–2.06)		1.15 (0.32–4.14)		0.31 (0.08–1.23)
**Smoking**					
No smoking		Ref.			
Current smoker		1.71 (0.80–3.64)			
Former smoker		1.66 (0.39–7.01)			

OR, Odds Risk; CI, Confidence Interval; Only significant variables (*p* < 0.05) in univariate analysis were considered for the five-stepwise binary logistic regression analysis, significant coefficients are shown in bold,

* *p* < 0.05,

** *p* < 0.01,

*** *p* < 0.001.

### Association between 5C psychological antecedents with COVID-19 vaccine acceptance

Table 4 summarizes the association between 5C psychological antecedents and willingness to accept COVID-19 vaccine. The respondents with a complacency was significantly associated with a high intention to receive a vaccine (OR = 3.97; 95% CI = 1.87–8.42, *p* < 0.001). On the other hand, the respondents with no collective responsibility showed low intention toward vaccine acceptance (OR = 0.23; 95% CI = 0.11–0.49, *p* < 0.001). Additionally, amid all covariates, gender and age, and perceived health condition were related to low intention to vaccine acceptance (OR = 0.22; *p* < 0.05, OR = 0.95; *p* < 0.05, and OR = 0.66; *p* < 0.05, respectively).

**Table 4:** Multinomial logistic regression results determine the association between 5C domains and willingness to accept the COVID-19 vaccine.

	**B**	**SE**	**Sig**.	**OR**	**95% CI**	
					**Lower bound**	**Upper bound**
Confidence (Ref. = Yes)	−0.392	0.365	0.282	0.67	0.33	1.38
Constraint (Ref. = Yes)	−0.553	0.378	0.144	0.57	0.27	1.21
Complacency (Ref. = Yes)	1.380	0.384	0.000^***^	3.97	1.87	8.42
Calculation (Ref. = Yes)	−0.522	0.339	0.123	0.59	0.31	1.15
Collective responsibility (Ref. = Yes)	−1.444	0.374	0.000^***^	0.23	0.11	0.49
Gen	−1.509	0.557	0.007^**^	0.22	0.07	0.66
Age	−0.052	0.017	0.003^**^	0.95	0.92	0.98
Marital	0.973	0.686	0.156	2.65	0.69	10.15
Edu	0.129	0.251	0.607	1.14	0.69	1.86
Occupation	0.107	0.110	0.333	1.11	0.89	1.38
Family type	0.405	0.593	0.494	1.50	0.47	4.79
Monthly income	−0.130	0.205	0.526	0.89	0.59	1.31
COVID-19 positive tested	0.656	0.700	0.349	1.93	0.49	7.59
BMI	0.049	0.053	0.350	1.05	0.95	1.17
Long-standing illness	0.211	0.434	0.627	1.24	0.53	2.88
Perceived health condition	−0.424	0.207	0.041^*^	0.66	0.44	0.98
Smoking status	−0.719	0.439	0.102	0.49	0.21	1.15
Childhood vaccination	0.708	0.393	0.071	2.03	0.94	4.38

SE, Standard Error; OR, Odds Risk; CI, Confidence Interval; Only significant variables (*p* < 0.05) in univariate analysis were considered for the multinomial logistic regression analysis, significant coefficients are shown in bold,

* *p* < 0.05,

** *p* < 0.01,

*** *p* < 0.001.

### ROC analysis of the 5C subscales

**Figure 5:**
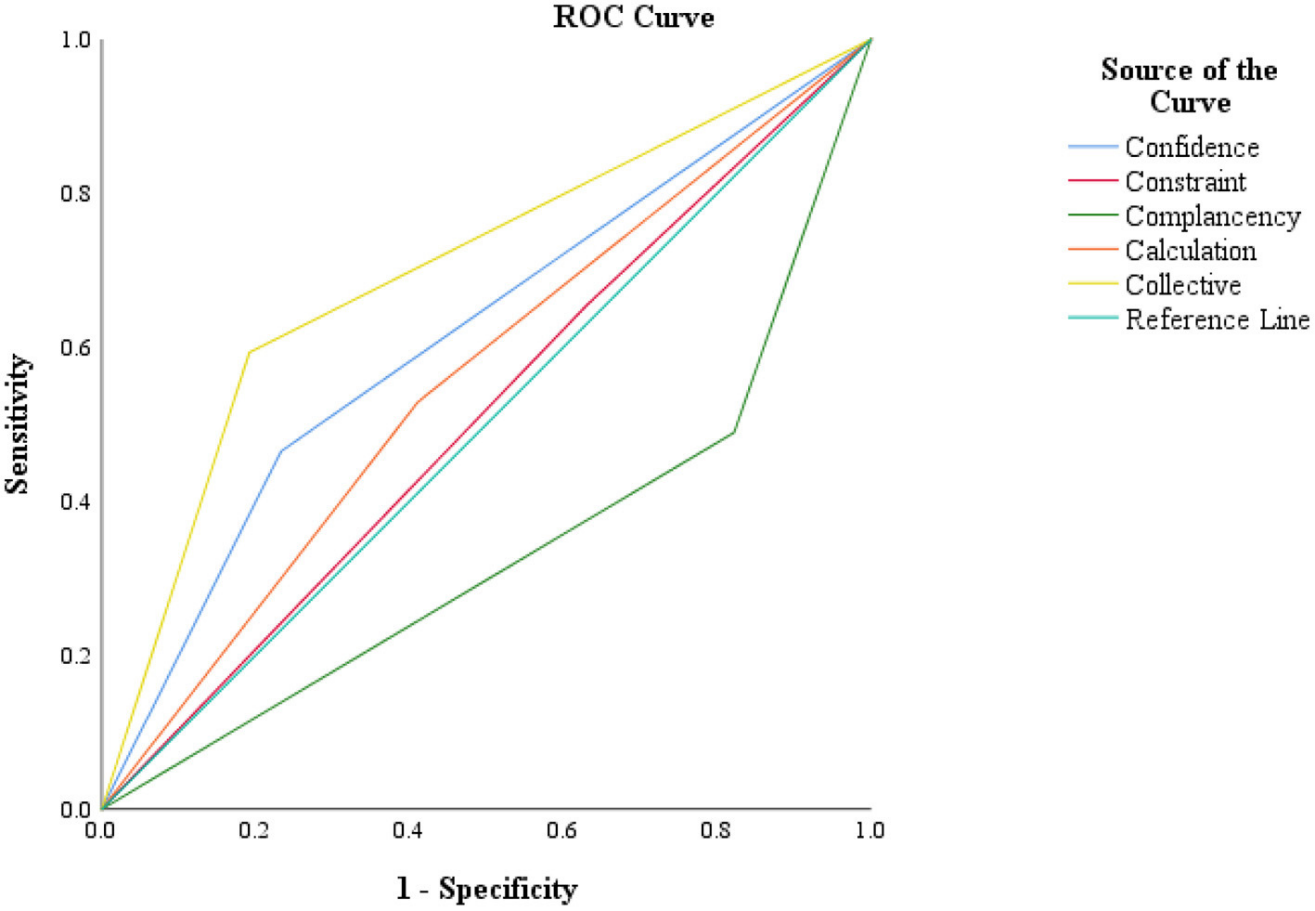
Receiver operating characteristic (ROC) analysis for 5C subscales in COVID-19 vaccine acceptance screening.

Figure 5 illustrates the ROC analysis of the 5C psychological antecedents. The ROC analysis disclosed that four domains, except complacency, appeared to be placed above the reference line. The highest AUC was found for collective responsibility (0.701). Beyond this, the AUC of confidence, calculation, and constraints were 0.616, 0.559, and 0.512, respectively. The lowest AUC was denoted in the case of the complacency subscale (0.334).

## Discussion

### Summary of the major findings

COVID-19 vaccine hesitancy has been globally a matter of concern (47). Despite multiple logistic efforts and national education programs, this issue continues to be a significant threat to the COVID-19 vaccine coverage in the coming days (10). In such a global scenario, the socially disadvantaged people, in particular, urban slum residents, are in vulnerable conditions to access vaccines. However, they should be prioritized for the COVID-19 vaccine as they are susceptible to infection because of their poor and unhygienic living condition. Understanding the psychological components that may impact an individual's attitude toward COVID-19 vaccination is crucial for generating evidence-based ways to minimize vaccine hesitancy (30). Given the dearth of research on psychological determinants of vaccine acceptance, this study explored the psychological determinants of COVID-19 vaccine acceptance among urban slum residents in Bangladesh.

The present study explored the psychological antecedents of COVID-19 vaccine acceptance among urban slum people of two large cities in Bangladesh using the 5C sub-scale. Our findings show that most of the slum dwellers who were confident, complacent, calculative, and responsible showed a higher vaccine acceptance rate. The slum residents those obtained vaccine information from the newspaper were highly willing to accept the COVID-19 vaccine. A high percentage of slum people who believe that coronavirus originated naturally and disagree with anti-vaccination were highly inclined to receive the COVID-19 vaccine. The regression results show that gender, marital status, education level, occupation status, monthly family income, long-standing illness, perceived health condition, and smoking behavior were significantly associated with 5C domains by different degrees. Further, two of the five psychological antecedents, including complacency and collective responsibility, were significantly associated with the vaccine acceptance rate to different degrees. Furthermore, the study found the highest AUC for the collective responsibility domain from ROC analysis.

Our results reveal that the majority of the slum residents had a greater level of confidence, calculation, and collective responsibility to be vaccinated. A similar finding was observed among migrants, another vulnerable population in Shanghai, China, where three in fourth respondents showed higher confidence levels in vaccine safety and effectiveness (48). In contrast, some constraints might be responsible for lessening the vaccine uptake, such as family dynamics and gender, geographical and technological barriers, and socioeconomic reasons (49). The respondents had a high level of calculation of information regarding the effectiveness and more details about the vaccine. The more people were found calculative, the more they hesitated toward vaccination (32). An earlier study in 13 Arab countries found that Sudan and Egypt had the highest calculation, which refers to assessing the benefits and risks of vaccination before making a decision (50). Amnesty International reported that the high calculation level is attributable to a lack of clear strategy and transparency for vaccination distribution, as well as insufficient vaccine information provided to local media and Egyptian authorities, and a limited awareness campaign (51). Our study also found a high level of collective responsibility, emphasizing the importance of herd immunity in controlling the spread of COVID-19. This thought increases the number of individuals willing to accept vaccines. Many recent studies have reported higher collective responsibility in line with our study findings (30, 32, 50, 52, 53).

Information sources also anticipated the acceptance of the COVID-19 vaccine. Three in fourth respondents supported TV or radio as the most common primary information source of vaccination information. However, vaccine acceptance seemed higher in the group that mentioned newspapers as a primary source. That might be for their high trust in the newspaper. One study conducted in Germany found that the participants who turned to the local newspaper for information were more likely to vaccinate, and the source positively affected vaccine intention (54).

Moreover, our study suggests that respondents who think the virus originated naturally had a greater acceptance rate for the COVID-19 vaccination. A similar finding was observed in research conducted in Kuwait, where more than 90 percent of healthcare professionals were favorable to vaccination acceptance and believed in the natural origin of COVID-19 (30). In addition, about eight out of every one hundred slum inhabitants in this survey were anti-vaccination, with over two-thirds demonstrating vaccine hesitancy. At the same time, approximately 90% of respondents who were not part of the anti-vaccination group anticipated high vaccine acceptance. These results were consistent with a prior study conducted in Kuwait (30).

In this study, the 5C psychological antecedents were influenced by the predictors, including being married, having primary and college-level education, earning 10,001–15,000 BDT or more per month, and having good health. A recent multinational study considering 13 Arab countries found that males, being of advanced age, educated, being a healthcare professional, having had COVID-19, or having an infected relative or one that died from COVID-19 as significant predictors regarding the 5C domains (50). Our study found that high-income slum people were less likely to be confident about vaccination. One possible explanation could be that aid from the government and non-government organizations might be a crucial factor in developing confidence in public authorities that affect residents' vaccination behavior (55). An earlier study also reported that higher trust and satisfaction in authorities were related to 1.95 times higher intention to be vaccinated (18).

Our findings suggest that people with higher income were less likely to have limitations toward vaccine uptake. In other words, low-income people were more likely to face restrictions on vaccination because of their loss of work hours or workdays. The majority of the respondents were workers and day laborers in our study. They need to earn daily to meet their daily needs, even a tiny amount (56). In addition, a study found that around 60% of those who received a second dose of the vaccine had various severe side effects, including fever, headache, myalgia, and general malaise (57). Fear of working days lost due to side effects of vaccination might impede the intention to vaccinate. Married participants in the present study were less likely to be calculative toward immunization. Their desire to vaccinate to protect their family members might make them less calculative. Our study found that primary and above college education and medium income levels were less likely to show complacency antecedents. A similar outcome was reported previously where people with post-graduate were less complacent (50). Furthermore, they believe economic and political uncertainty may contribute to people's complacency with vaccines. Finally, people with a high level of family income and good health showed varying levels of collective responsibility. In this research, the high-income group was positively related to collective responsibility. People with fair health, as opposed to very good health, were less inclined to consider collective responsibility. Respondents with fair, poor, or very poor health may be concerned about the side effects of vaccination rather than considering collective responsibility.

Low levels of complacency and high levels of collective responsibility were linked with COVID-19 vaccination acceptability among the slum dwellers. Prior research on Bangladeshi adults supported these results (32). This research found that more collective responsibility considerably decreased vaccination hesitancy, but greater complacency significantly increased vaccine hesitancy. Conversely, reduced complacency and more collective responsibility were positively related to high vaccination intent seen among nurses (52). Individuals with a complacent attitude usually believe that vaccination is unnecessary since their immune systems are capable of protecting them from infection. It was observed that the Chinese thought they did not need to be vaccinated since they were physically well, which affected their intention to get vaccinated (58).

The ROC analysis for all of the psychological domains in this study suggests that four of the 5C sub-scales, with the exception of complacency, might be useful in predicting COVID-19 vaccine uptake. Similar results have been found among Kuwaiti healthcare professionals, with the exception of the math sub-scale. Similar findings have been reported in healthcare workers of Kuwait; however, their exception was for the calculation sub-scale (30).

### Implications of the study

The notable implication of this study is that the application of 5C psychological antecedents would assist in understanding the confidence, complacency, constraints, calculation, and collective responsibility of slum dwellers toward COVID-19 vaccine acceptance. Beyond this, sociodemographic predictors significantly affect this 5C and are a solid addition to this study. While vaccine development and availability are essential to accomplish herd immunity against the pandemic, the study will assist local public health representatives design targeted vaccine intervention programs regarding vaccination coverage successes. Recognizing the variables and determinants of COVID-19 vaccine uptake would help increase the efficiency of these rollout campaigns.

### Strength and limitations of the study

This study investigated the COVID-19 vaccine acceptance among the slum people of two large cities in Bangladesh, using a large and diverse representative sample. Slum people are considered a backward community because of their socioeconomic vulnerability (40). Therefore, exploring their intention to vaccination will be an efficient addition to public health concerns. Moreover, a significant strength of this study was adopting the 5C sub-scale for evaluating the psychological determinants of vaccinations. The scale has an admissible discriminatory power with its identified cutoff score to anticipate the psychological antecedents regarding COVID-19 vaccine acceptance (59). However, there are some limitations to this study. We could not draw causal connections between variables of interest because of the cross-sectional study design. However, the association between psychological antecedents and vaccine acceptance can be tracked over time in longitudinal studies, which may help researchers determine how health-related policies affect these factors. Besides, the study's findings were based on self-reported data that introduced information bias.

Further, our sample is disproportionately female since most study participants were housewives found in their homes at the time of data collection instead of the income person. Finally, we considered only two large cities, including the capital city; however, we could not include the slum areas of the entire country. The nationwide representative samples should be focused on in future research.

## Conclusions and recommendations

The study investigated the psychological antecedents of COVID-19 vaccination acceptability among slum dwellers in two Bangladeshi cities. Vaccine acceptance was higher among slum inhabitants who were confident, complacent, calculated, and collectively responsible. Further, individuals who received information from the newspaper were more inclined to accept the COVID-19 vaccine. Similarly, more significant percentages of slum dwellers believed coronaviruses were naturally occurring and refused to get vaccinated. Marital status, education, family income, and perceived health condition significantly predicted the 5C domains. Two antecedents, complacency, and collective responsibility, were significantly associated with vaccine acceptance.

These results might assist policymakers in developing appropriate measures for increasing vaccine acceptance among the urban slum population of Bangladesh. Government activities and laws, the media, and healthcare organizations should play a vital role in influencing the public's attitude regarding COVID-19 vaccinations to maximize vaccination acceptance. To convince the public to vaccinate against COVID-19, several social actors, the great majority of whom are often marginalized from mainstream politics and health policy, would need to collaborate. In addition, vaccination reluctance might be reduced with an effective communication campaign that debunks COVID-19 vaccination conspiracy theories. This may be achieved by highlighting the need to communicate clear information *via* reliable sources (e.g., scientists and scientific journals) and fact-checking the statements made on television, newspapers, and social media platforms. Finally, the Government of Bangladesh should initiate public health education programs among the urban slum population to increase their basic health literacy, with a larger focus on the perception of vaccination benefits and disease severity.

## Data availability statement

The raw data supporting the conclusions of this article will be made available by the authors, without undue reservation.

## Ethics statement

The studies involving human participants were reviewed and approved by the Research Ethical Clearance Board of the Institute of Disaster Management, Khulna University of Engineering & Technology, Khulna, Bangladesh. Written informed consent for participation was not required for this study in accordance with the national legislation and the institutional requirements.

## Author contributions

MP: conceptualization, methodology, formal analysis, writing—original draft, review and editing. MB: conceptualization, survey development, data curation, and writing—original draft. SA: conceptualization, survey development, and writing—original draft. MeH, FI, SR, MN, and MaH: conceptualization, survey development, data collection, and data curation. MZH and AD: conceptualization and survey development. MRH, AR-M, FS, SN, and SS: writing—review and editing. All authors contributed to the article and approved the submitted version.

## Conflict of interest

The authors declare that the research was conducted in the absence of any commercial or financial relationships that could be construed as a potential conflict of interest.

## Publisher's note

All claims expressed in this article are solely those of the authors and do not necessarily represent those of their affiliated organizations, or those of the publisher, the editors and the reviewers. Any product that may be evaluated in this article, or claim that may be made by its manufacturer, is not guaranteed or endorsed by the publisher.

## Abbreviations

COVID-19: Coronavirus Disease 2019
EPI: Expanded Programme on Immunization
ROC: Receiver Operator Characteristic
LMIC: Lower-middle Income Countries
BMI: Body Mass Index.
